# Alterations in lipid profile upon uterine fibroids and its recurrence

**DOI:** 10.1038/s41598-021-89859-0

**Published:** 2021-06-01

**Authors:** Narine M. Tonoyan, Vitaliy V. Chagovets, Natalia L. Starodubtseva, Alisa O. Tokareva, Konstantin Chingin, Irena F. Kozachenko, Leyla V. Adamyan, Vladimir E. Frankevich

**Affiliations:** 1 National Medical Research Center for Obstetrics, Gynecology and Perinatology named after Academician V.I. Kulakov of the Ministry of Healthcare of Russian Federation, Moscow, 117997 Russian Federation; 2 V.L. Talrose Institute for Energy Problems of Chemical Physics, Russia Academy of Sciences, Moscow, 119991 Russian Federation; 3 Moscow Institute of Physics and Technology, Moscow Region, 141700 Russian Federation; 4 Jiangxi Key Laboratory for Mass Spectrometry and Instrumentation, East China University of Technology, Nanchang, 330013 China

**Keywords:** Diagnostic markers, Predictive markers, Prognostic markers, Molecular medicine

## Abstract

Uterine fibroids (UF) is the most common (about 70% cases) type of gynecological disease, with the recurrence rate varying from 11 to 40%. Because UF has no distinct symptomatology and is often asymptomatic, the specific and sensitive diagnosis of UF as well as the assessment for the probability of UF recurrence pose considerable challenge. The aim of this study was to characterize alterations in the lipid profile of tissues associated with the first-time diagnosed UF and recurrent uterine fibroids (RUF) and to explore the potential of mass spectrometry (MS) lipidomics analysis of blood plasma samples for the sensitive and specific determination of UF and RUF with low invasiveness of analysis. MS analysis of lipid levels in the myometrium tissues, fibroids tissues and blood plasma samples was carried out on 66 patients, including 35 patients with first-time diagnosed UF and 31 patients with RUF. The control group consisted of 15 patients who underwent surgical treatment for the intrauterine septum. Fibroids and myometrium tissue samples were analyzed using direct MS approach. Blood plasma samples were analyzed using high performance liquid chromatography hyphened with mass spectrometry (HPLC/MS). MS data were processed by discriminant analysis with projection into latent structures (OPLS-DA). Significant differences were found between the first-time UF, RUF and control group in the levels of lipids involved in the metabolism of glycerophospholipids, sphingolipids, lipids with an ether bond, triglycerides and fatty acids. Significant differences between the control group and the groups with UF and RUF were found in the blood plasma levels of cholesterol esters, triacylglycerols, (lyso) phosphatidylcholines and sphingomyelins. Significant differences between the UF and RUF groups were found in the blood plasma levels of cholesterol esters, phosphotidylcholines, sphingomyelins and triacylglycerols. Diagnostic models based on the selected differential lipids using logistic regression showed sensitivity and specificity of 88% and 86% for the diagnosis of first-time UF and 95% and 79% for RUF, accordingly. This study confirms the involvement of lipids in the pathogenesis of uterine fibroids. A diagnostically significant panel of differential lipid species has been identified for the diagnosis of UF and RUF by low-invasive blood plasma analysis. The developed diagnostic models demonstrated high potential for clinical use and further research in this direction.

## Introduction

Uterine fibroids (UF), also referred to as myomas, is the most common (about 70% cases) type of gynecological disease^1–6^. 25% of UF patients of reproductive age undergo surgery^1–3^. UF has no distinct symptomatology and is often asymptomatic, which makes it difficult to diagnose^1,2^. The common complaints of patients with UF include painful and heavy menstruation, abnormal uterine bleeding, pain in the lower abdomen, infertility, miscarriage, impaired function of adjacent organs, dyspareunia, etc.^7–9^.

The pathogenesis of UF remains unknown. The roles of genetic mutations^10,11^, hormonal disorders (estrogen-progesterone imbalance), neoangiogenesis^12^, and growth factors^13^ have been reported^14^. Risk factors of UF include early menarche, late reproductive age, obesity, high parity or nulliparity, menopause, smoking, combined oral contraceptives, inflammatory processes^1,6,12,15–17^.

For the UF patients who are planning pregnancy, myomectomy remains the main treatment. However, the recurrence of UF is possible after organ-preserving treatment. The recurrence rate of UF varies from 11 to 40%. A second surgery is necessary in 1.3–27% of cases^18^. UF belongs to diseases with a genetic predisposition^19^. A family history of UF was shown to increase the risk of UF recurrence^19,20^. Risk factors for UF recurrence include the presence of more than 3 fibroids, age from 30 to 40 years, rapid tumor growth before surgery, as well as certain histological types of UF^21^. Surgery can trigger the development of new myomatous nodes, because damage to the myometrium activates the expression of mitogenic and angiogenic growth factors. High level of Ki-67 (Ki-67), progesterone receptor (PgR) and vascular endothelial growth factor (VEGF) in the myometrium and fibroids are pathogenetic factors of UF recurrence^22–24^.

Currently, there is a lack of specific (laboratory, instrumental) criteria for the prediction of UF recurrence. Reliable prediction of UF recurrence would help the practicing physician to determine the required amount of surgical treatment, minimize risks of repeated surgical interventions, and increase the effectiveness of organ-preserving treatment^25^.

The search of new UF biomarkers is mainly done by metabolomics and proteomics approaches, because these approaches allow determination of the molecular composition for any biological sample with high accuracy^26^. Shotgun lipidomics based on electrospray ionization mass spectrometry (ESI–MS) allows deep molecular profiling of a sample without significant losses of chemical information^27,28^. The high diagnostic potential of lipidomics has been shown in many areas of medicine, particularly in oncology: lung, thyroid gland, breast, stomach, pancreas, colorectal, liver, kidney, prostate, ovarian, and endometrium cancer^29–52^. MS studies of lipid profiles in tissues and blood plasma have revealed new promising biomarkers of endometriosis (benign gynecological disorder)^27,28,37,40,47,48,52^. To date there are no sufficient metabolomics data for uterine fibroids, as only one study by Heinonen H. R. group was conducted in tissues^53^. Heinonen et al. found that homocarnosine level was reduced in all fibroid subtypes studied; sphingolipids, phosphatidylserines, vitamin A and C levels were reduced in MED12 mutated fibroids^53^. A significant decrease in the level of lipids in the tumor tissue may lead to a small size of subtype MED 12 UF^53,54^. A comparative MS study of lipid profiles of blood plasma, tissues of fibroids and myometrium may reveal new molecular markers for the diagnosis and prediction of the course of UF, in particular, access the risk of UF recurrence.

This study aimed at evaluating the potential of the lipid profiling of blood plasma for the low-invasive diagnosis of fibroids recurrence, which is important to choose adequate surgical treatment as well as to improve the efficiency of reconstructive plastic surgeries.

## Materials and methods

### Study design

The study of fibroids and myometrium tissues included 35 women with uterine fibroids (UF) diagnosed for the first time and 31 patients diagnosed with recurrent uterine fibroids (RUF).

For the group of first-time diagnosed UF, patients with the absence of anamnestic risk factors for recurrence were selected (a small number of nodes—1–2 nodes, the absence of a familial form of UF).

Also, during the observation period after surgical treatment (3.5–4 years), patients from this group were not diagnosed with a relapse. A control group with a matched age for a comparative analysis of blood plasma included 15 patients operated for infertility and for the intrauterine septum. Patients from the control group had no UF, both according to ultrasound and laparoscopy data. All patients (n = 81) were examined in the department of Operative Gynecology of National Medical Reseach Center for Obstetrics, Gynecology and Perinatology named after Academician V.I. Kulakov of the Ministry of Healthcare of Russian Federation. All patients signed an informed consent to participate in the study, approved by the Ethics Committee of National Medical Reseach Center for Obstetrics, Gynecology and Perinatology named after Academician V.I. Kulakov. We confirm that all methods were performed in accordance with the relevant guidelines and regulations.

Inclusion criteria for the UF and RUF groups were: reproductive age (18–45 years), uterine fibroids, organ-preserving surgery, lack of hormone therapy for 6 months or more before surgery. Exclusion criteria were: systemic autoimmune diseases, severe somatic pathology, cancer, inflammatory processes, concomitant gynecological pathology. All patients underwent organ-preserving treatment with endoscopic access over the first phase of their menstrual cycle. The indications for surgical treatment were heavy menstruation leading to anemia, severe pain syndrome, lack of effect from previous conservative therapy, and infertility.

### Sample collection

Myometrial and fibroids samples were collected during surgery. Samples of myomatous nodes were obtained from the largest node from the central part. Tissue samples were placed in a sterile cryovials (Corning), transported in liquid nitrogen to a Biobank, and stored in a freezer at the temperature of − 80 °C until analysis. Blood sampling was performed on an empty stomach on the eve of surgery. Blood was collected in a sterile vacuum tube with EDTA-sodium and centrifuged for 10 min at 2500 rpm to obtain plasma. Plasma was stored in sterile cryovials (Corning) in a freezer at − 80 °C until analysis.

### Sample preparation for lipidome analysis

Plasma and tissue lipid extracts were prepared according to the modified Folch method^27,28,55^. Briefly, after homogenization of 50 mg of tissue in liquid nitrogen, 5 μL of internal standard and 4 mL of a chloroform–methanol (2:1, v/v) were added, incubated for 10 min, and filtered. Then, 800 μL of 1 M NaCl solution in water was added and centrifuged. An organic layer containing lipids was collected, vacuum dried, and redissolved in 500 μL 2-propanol-acetonitrile (1:1,v/v) for MS analysis.

For plasma samples, 480 μL and 5 μL of internal standard l of chloroform–methanol (2:1,v/v) was added to 40 μL of a plasma. The mixture was sonicated for 10 min. Then, 150 μL of H_2_O was added. The mixture was centrifuged for 5 min at 15,000 rpm at ambient temperature. An organic layer was collected, vacuum dried and then redissolved in 200 μL 2-propanol-acetonitrile (1:1, v/v) for MS analysis.

Equal amounts of all samples were pooled as a QC sample for MS system conditioning and quality control.

### Mass spectrometry analysis of lipid extracts

The molecular composition of tissue lipid extracts was determined using electrospray ionization mass spectrometry (ESI–MS) on a Maxis Impact qTOF mass spectrometer (Bruker Daltonics, Bremen, Germany). Mass spectra were obtained in both positive and negative ion detection modes in the *m/z* range of 100–1800 with the following settings: 4.1 kV capillary voltage in positive ion mode (3.0 kV in negative ion mode), spray gas pressure 0.7 bar, drying gas flow rate 6 L/min, the temperature of the drying gas 200 °C^27,28^.

The molecular composition of plasma lipid fraction was determined by HPLC–MS using a Dionex UltiMate 3000 liquid chromatograph (Thermo Scientific, Germany) connected to a Maxis Impact qTOF mass analyzer with an ESI ion source (Bruker Daltonics, Germany). Lipids were separated by reverse phase chromatography on a Zorbax C18 column (150 × 2.1 mm, 5 μm, Agilent, USA) with a linear gradient of 30% to 90% eluent B (acetonitrile/2-propanol/water, 90:8:2, v/v/v, with 0.1% formic acid and 10 mM ammonium formate) in 20 min. Acetonitrile/water (60:40, v/v) with of 0.1% formic acid and 10 mM ammonium formate was used as eluent A. The elution flow rate was 40 μL/min. The volume of the injected sample was 3 μL. Mass spectra were obtained in the positive ion mode over the mass range *m/z* 400–1000 with resolution of 50,000 and the following ion source settings: capillary voltage 4.1 kV, spray gas pressure 0.7 bar, drying gas flow rate 6 L/min, the temperature of the drying gas is 200 °C. Quality control samples were injected randomly between the samples and used to evaluate the quality of our experiments.

Tandem MS analysis (MS/MS) was done using data dependent analysis mode. Five the most abundant peaks were chosen after full MS scan and subjected to MS/MS analysis (CID) with 35 eV collision energy, 3 Da isolation window and mass exclusion time of 1 min.

### Statistical analysis

Lipids from myometrium and fibroids tissues were identified with in-lab created R code (the RStudio version was 1.1.463 and the R language version was 3.5.2) by exact mass within 10 ppm mass accuracy using the theoretical computer-generated database of mass lipids for a given ion, class, total length of fatty acid residues and characteristic tandem mass spectra (MS/MS). Blood plasma lipids were identified using the Lipid Match R-script^56^ for the exact mass within 10 ppm mass accuracy^57^ and for the tandem mass spectra (MS/MS).

Statistical significance of lipid level changes between UF and RUF in myometrium and fibroids and between control and UF, control and RUF, UF and RUF in plasma was studied by a non-parametric two-way Mann–Whitney U-test (p < 0.05). To determine the metabolic pathways enriched in uterine fibroids, lipid, with significant differences in tissue and plasma, were analyzed by the online resource Metaboanalyst 4.0 (https://www.metaboanalyst.ca/) using hypergeometric test methods and KEGG library for Homo Sapience.

The classification models for control and UF, control and RUF, UF and RUF were built using the discriminant analysis method with orthogonal projection on latent structures (OPLS-DA) for lipids with a significant difference in levels. Quality of the PLS-DA model was estimated by R^2^ and Q^2^ values. Q^2^ was calculated by sevenfold leave-one-out cross-validation (LOOCV). Potential lipid markers included lipids with the greatest importance of the independent variable for projection (VIP) values according to the OPLS-DA model (VIP > 1). The selected lipids were used for creation diagnostic models based on logistic regression with the formula $y=\frac{1}{1+{e}^{-({\beta }_{o}+\beta \times {I}^{t})}}$, where y is variable response with values 0 in cases of control group and 1 in cases of myoma, ${\beta }_{o}$ is free coefficient, β is vector of coefficients, and I is the vector of marker’s intensity. Sensitivity and specificity of the models were evaluated by leave-one-out cross-validation^58–60^.

## Results and discussion

### Clinical data

The study included 81 women of reproductive age and Caucasian race divided into three groups. The first group included 35 women with uterine fibroids (UF) diagnosed for the first time. The second group included 31 patients diagnosed with recurrent uterine fibroids (RUF). The third control group included 15 patients operated for infertility and for the intrauterine septum.

**Figure 1:**
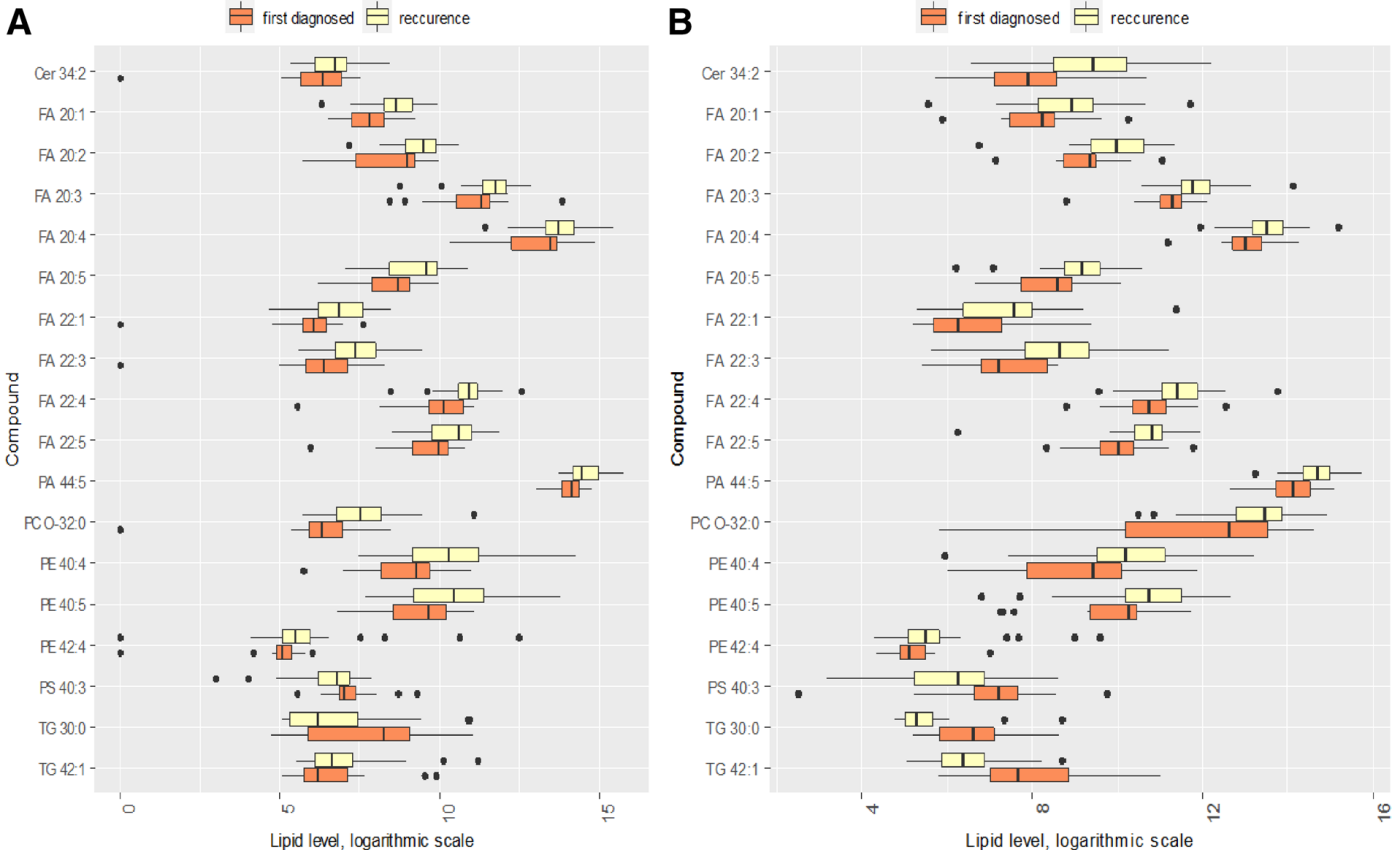
Relative intensity of marker lipids (p < 0.05) in the mass spectrum of (**A**) myometrium and (**B**) uterine fibroids. Orange color corresponds to the first-time diagnosed UF. Yellow color corresponds to RUF. The diagram shows Q1 − 1.5 × IQR, Q1, Me, Q3, Q3 + 1.5 × IQR. Black dots correspond to outliers. *Cer* ceramides, *FA* fatty acids, *PC-O* plasmalogens, *PA* phosphotidyl acids, *PE* phosphotidylethanolamines, *PS* phosphotidylserines, *TG* triacylglycerides.

The patients included in the study were of reproductive age (more than 80% were 36–45 years old). The average age of patients with UF 37.6 ± 5.5 years, and patients with RUF—39.8 ± 5.9 years (Table 1). The clinical diagnosis of patients was done on the basis of an objective examination, ultrasound data and finally verified according to the data of histological examination (Fig. 1S). All patients underwent organ-preserving treatment with endoscopic access over the first phase of their menstrual cycle.

**Table 1:** Clinical and demographical data for UF and RUF patients.

	First-time diagnosed uterine fibroids (n = 35)	Recurrent uterine fibroids (n = 31)	p-value
Age, years	37.6 ± 5.5	39.8 ± 5.9	> 0.5
Body mass index	24 ± 5.0	25 ± 4.0	< 0.5
Menarche, years	12.9 ± 1.0	12.8 ± 1.3	> 0.5
Menstrual cycle length, days	27.8 ± 2.4	27.6 ± 3.1	> 0.5
Duration of menstruation, days	5.4 ± 1.2	5.3 ± 1.4	> 0.5
Number of pregnancies	1.3 ± 1.8	1.3 ± 1.5	> 0.5
Infertility complaints, %	21.3	31.9	< 0.5
Uterine fibroids in close relatives, %	49.2	56.45	< 0.05
Duration of surgery, min	94.6 ± 39.4	122.7 ± 61.4	< 0.001
Blood loss, mL	234.7	299.2	< 0.001
Duration of infertility, years	5.6 ± 4.4	7 ± 4.5	< 0.05
Size of the main node, cm	8.2 ± 4.3	6.5 ± 3.8	< 0.5
Number of removed nodes	1 ± 1	1 ± 1	< 0.5
Submucous fibroids (type 1, 2, FIGO), %	9.8	22.7	< 0.5
Interstitial-submucous fibroids (type 1,2, FIGO), %	11.2	22.7	< 0.5

We discovered that the first myomectomy was performed in the age from 36 to 41 years and the myomectomy caused by RUF was performed in the age from 42 to 45 years. Significant (p < 0.039) excess of BMI was observed in patients with RUF. Pain syndrome and problems with the onset and bearing of pregnancy were most pronounced in the RUF group. Patients from the UF group complained of infertility with an average duration of 5.6 ± 4.4 years. In the RUF group, infertility occurred in 31.9% of cases with an average duration of 7 ± 4.5 years.

Diabetes and uterine fibroids are significantly more frequent in the closest relatives (p < 0.05) for UF and RUF groups compared to control group. The data obtained confirm the presence of a family predisposition of UF. The frequency of detection of submucous (MM 9.8%, PMM 22.7%), and interstitial-submucous (MM 11.2%, PMM 22.7%) fibroids (type 0, 1, 2, FIGO) during ultrasound examination is higher for RUF group compared to UF group. Similar data were obtained by assessing the intraoperative localization of nodes. This observation can be explained by the presence of hormonally active tissue near the endometrium and the result of the previous operation (reduction of myometrial tissue and growth of fibroids towards the uterine cavity).

For RUF, a long duration of surgical treatment was observed. This indicates the complexity of the repeated organ-preserving surgery, considerable intraoperative blood loss and more frequently used reinfusion of erythrocytes. In the RUF group, a greater number of myomatous nodes were removed (MM—1 ± 1 nodes, RUF—5 ± 5 nodes, p < 0.5). However, the size of the removed nodes prevailed in the UF group (UF—8.2 ± 4.3, RUF—6.5 ± 3.8, p < 0.5).

Surgical interventions were performed in 2017–2018. No recurrences were detected in the group of first-time diagnosed UF for 3.5–4 years. The patient’s data is monitored (including ultrasound control 2 times a year) in National Medical Reseach Center for Obstetrics, Gynecology and Perinatology named after Academician V.I. Kulakov. The percentage of recurrence in the group of RUF was 15.8%, after 12 months and 31.2% after 24 months.

During the follow-up period after myomectomy over 12–18 months, pregnancy occurred in 9.7% of cases in the group of first recurrence (second-time diagnosed UF) and in 34.2% of cases in the group of first-time diagnosed UF.

In parallel with tissue profiling, morphological analysis (including immunohistochemical examination of myoma and myometrium tissues) was also performed. It was found that the expression of VEGF is higher in tumor tissue compared to myometrium samples from patients with MM and PMM. The Ki-67 level is higher in myomatous nodes in patients with PMM (p = 0.031), which may reflect the proliferative potential of the tumor most susceptible to recurrence. The expression of ER and PgR (p = 0.012) is higher in the tissue of myomatous nodes in patients with PMM, which reflects the potential of tumor growth. Thus, for the selection of first-time diagnosed UF into the group, we also focused on the low expression of the above markers.

### Uterine myometrium and fibroids lipidomics (ESI–MS/MS)

The total of 296 lipid species was identified in the tissue of the myometrium and fibroids. Out of the 296 identified lipid species, 66 lipid species showed statistically significant abundance variation between the diagnosed UF and its recurrence in the tissues of the fibroids and 39 lipid species showed statistically significant abundance variation between the diagnosed UF and its relapse in the tissues of the myometrium (Tables S1, S2). Level of all significant different ceramides, sphingomyelins, fatty acid, phosphatidylethanolamines and phosphatidic acids increased in tissue in RUF group compared to UF. In addition, level of larger part of significantly different phosphatidylcholines and triacylglycerol TG 48:4 increased. Level of all significantly different phosphatidylserines, phosphatidylcholines PC 32:3 and PC 46:0 and larger part of triacylglycerol decreased in recurrence fibroids. Thus, the greatest alterations in the lipid composition during fibroids recurrence were observed in the tumor tissue. The level of 20 lipid species changed significantly both in the myometrium and in the myomatous nodes during the disease recurrence (Fig. 1). For 19 out of these 20 species the increase in expression was found.

**Figure 2:**
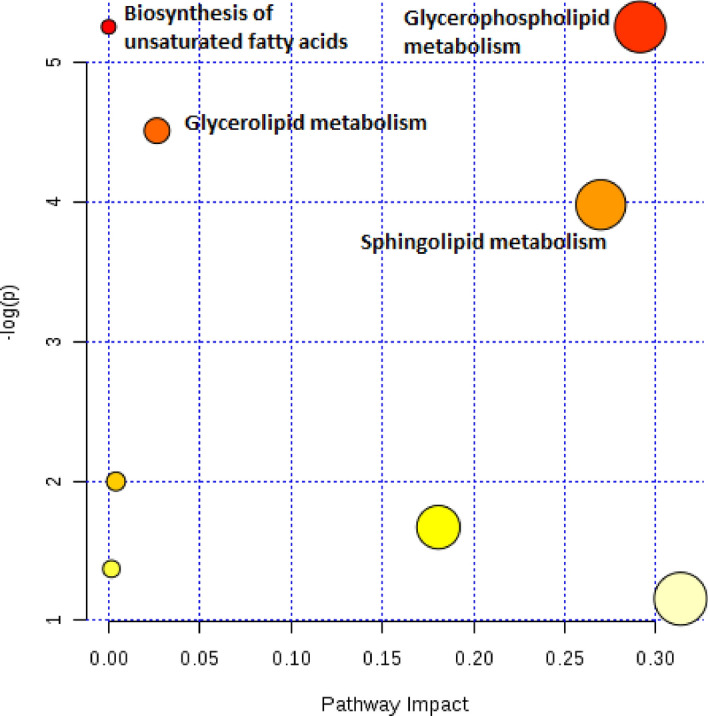
Diagram of metabolic pathways for lipid species with statistically significant abundance variation common for benign tumor and myometrium during recurrence of fibroids.

Enrichment of the linoleic acid, glycerophospholipids, ether lipids, sphingolipids metabolism was shown for benign tumor tissue during recurrence of UF (Fig. 2S). Differential lipid species that are statistically significant for both myometrium and fibroids were found to be mainly involved in the metabolism of glycerophospholipids and sphingolipids (Fig. 2). This indicates the similarity of metabolic processes for myometrium and fibroids during recurrence of fibroids. In contrast, linoleic acid metabolism undergoes changes only in UF cells. Differences in the metabolism of linoleic acid in UF cells compared with myometrial cells, as well as a changes in the fatty acid profile of the cells were previously noted by Islam and Castellucci^61^.

In this study, plasmalogens (PC-O and PE-O) were significantly (p < 0.05) elevated in myometrium and fibroid tissue in recurrent form of uterine myoma. Ether-phospholipids and their metabolites are involved in protein kinase C (PKC) signalling cascades^62^.

We observed that phosphotidyl acid PA 44:5 was significantly higher in both miometrium and fibroid tissue in RUF group. This is the first evidence that PA is involved in the pathophysiology of UF. PA is a phospholipid that consists of a glycerol backbone with two fatty acids and one phosphate group attached, which is a central intermediate in the synthesis and storage of membrane lipids^63^. PA has been involved in various cellular signaling pathways, including cell growth, proliferation, cell motility, and the production of reactive oxygen species^63^. PA has been shown to have anti-apoptotic effects^63^. Also, PA has been identified as a mitogenic activator of the mammalian target of rapamycin signaling pathway to promote cell proliferation and generate survival signals^64^ This might contribute to active proliferative capacity of UF. Moreover, PA is related to cell motility^65,66^, which may promote migration and invasion of UF cells.

Sphingomyelins were abundant in recurrent uterine fibroids, promoting cell survival in response to apoptotic stimuli^67^ Hydrolysis of sphingomyelins results in ceramides release. We found that the level of ceramides (Cer) was also increased in myometrium and fibroid tissue upon RUF. Moreover, Cers are known to be signaling molecules related to inflammation and apoptosis^68^.

The level of phospatidylcholines upon RUF changed in both directions: the level of six PCs (PC 34:0, PC 34:1, PC 36:1, PC 36:2, PC 36:4, PC 38:2) was significantly elevated, and the level of three PCs (PC 32:3, PC 46:0, PC 48:5) was decreased. PC is known to be one of the major sources of polyunsaturated fatty acids (FA), which serve as the precursors of eicosanoids and have numerous biological activities^69^ The level of FAs was also increased upon RUF. PCs contribute to both proliferative growth and programmed cell death^70^. The synthesis of PC is enhanced in response to FA and FA-derived substrates, which is frequently observed in cancer cells^70^.

Phosphatidylserines (PS 38:3 and PS 40:3) were significantly decreased in fibroids of RUF group. This result is consistent with the results of the study by Heinonen et al.^53^. The exposure of PS to the cell surface shows an apoptotic signal for phagocytes^71^. Lower PS levels may be due to reduced UF apoptotic cells in RUF cases.

Thus, RUF is associated with elevated tissue levels of sphingomyelins, ether-phospholipids, phosphotidyl acids, sphingomyelins, ceramides, which might contribute to the suppression of apoptosis, promotion of cell proliferation and affect lipid-associated signaling pathways.

### Blood plasma lipidomics in recurrent uterine fibroids (HPLC–MS/MS)

**Figure 3:**
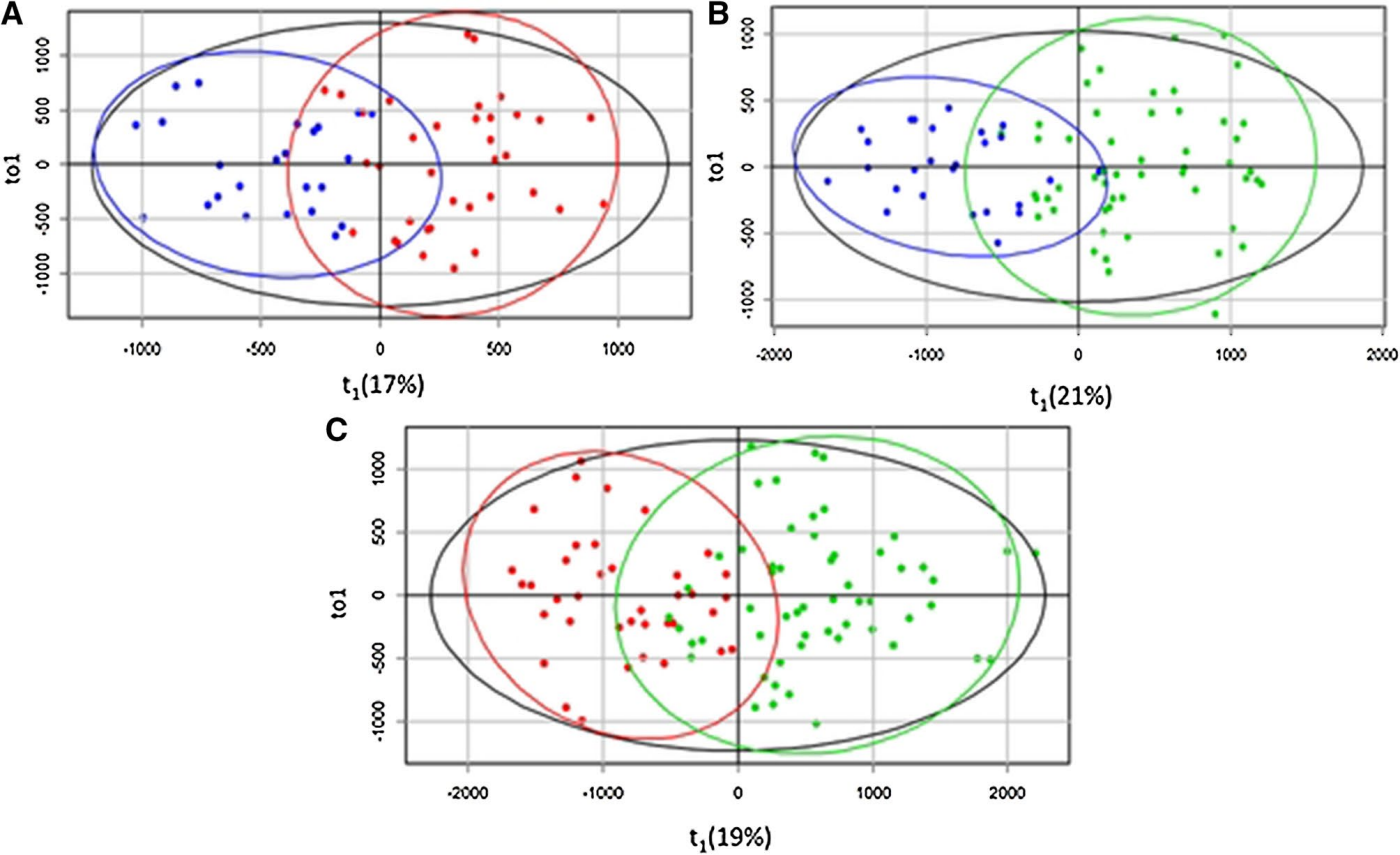
OPLS-DA score plots of plasma lipidomic data (blue dots correspond to control group, red dots correspond to the UF group, and green dots correspond to RUF): (**A**) Control group vs. first-time diagnosed UF. (**B**) Control group vs. RUF. (**C**) First-time diagnosed UF vs. RUF.

The total of 267 lipid species was identified in blood plasma samples. The lipid levels were tested by pairwise Mann–Whitney U-test: “control group vs. first-time diagnosed fibroids”, “control group vs. recurrent fibroids” and “first-time diagnosed fibroids vs. recurrent fibroids”. Statistically significant differences were found for 43 lipid species in the first case (control vs. first-time UF), 64 in the second case (control vs. RUF) and 87 for the third case (first-time UF vs. RUF). OPLS-DA models were constructed to classify patients (Fig. 3).

For the OPLS-DA models distinguishing between control group and UF group (Fig. 3A) and between control group and RUF group (Fig. 3B), 70% and 67% of data were included (R^2^Y). The expected classification accuracy for new samples (Q^2^Y) was 63% and 60%, accordingly. The values of R^2^Y > 50% and Q^2^Y > 40% suggest that there are significant changes in the lipid profile of blood plasma upon UF. For the OPLS-DA model distinguishing between UF and RUF groups, parameters R^2^Y and Q^2^Y were equal to 61% and 47%, respectively (Table 2). Thus, our data indicate that the recurrence of fibroids is accompanied by significant changes in lipid metabolism in the whole body.

**Table 2:** The parameters of OPLS-DA models.

	Lipids with VIP > 1	R^2^X	R^2^Y	Q^2^Y
Control group vs first-time diagnosed UF	LPC 18:2, PC 16:0_20:3, PC 18:0_18:1, PC 18:0_20:3, SM d18:1/22:0, SM d18:1/22:1, SM d18:1/24:0, TG 18:0_18:1_18:1	0.49	0.70	0.63
Control group vs RUF	PC 16:0_22:6, PC 16:0_18:2, PC 16:0_20:3, PC 18:0_20:3, PC 18:0_18:1, SM d12:0/14:1, SM d18:1/24:1, SM d18:2/24:1	0.36	0.67	0.60
First-time diagnosed UF vs RUF	CE 18:2, CE 20:4, PC 16:0_22:6, PC 18:0_18:2, SM d12:0/14:1, SM d18:1/22:0, SM d18:1/22:1, SM d18:1/24:0, SM d18:1/24:1, SM d18:2/16:0, SM d18:2/24:1, TG 14:1_18:1_18:2, TG 16:0_16:1_18:2, TG 16:0_18:1_18:2, TG 16:1_18:0_18:1, TG 16:1_18:0_18:3, TG 18:1_18:2_18:3	0.32	0.61	0.47

*CE* cholesterol esters, *LPC* lysophosphatidylcholines, *PC* phosphatidylcholines, *SM* sphingomyelins, *TG* triglycerides.

The largest contribution (VIP > 1) to the differentiation between the control group and the UF group was provided by phosphotidylcholines and sphingomyelins. Three lipid species, including PC 16:0_20:3, PC 18:0_20:3 and PC 18:0_18:1, were significantly decreased in the blood plasma of UF patients compared to control group (Figs. S3, S4).

Diagnostic models based on the selected lipid species using logistic regression (Tables 3, 4) show sensitivity and specificity of 88% and 86% for the diagnosis of first-time UF and 95% and 79% for the diagnosis of RUF. These results indicate the potential suitability of the lipid profiling of blood plasma for the low-invasive diagnosis of uterine fibroids.

**Table 3:** Coefficients for logistic regression of diagnostic model “control group/first-time diagnosed UF”.

	β	CI β	Z stat	p
Free coefficient	3.49E1	1.84E1 to 6.07E1		
LPC 18:2	− 6.46E−6	− 2.22E−5 to 7.94E−6	− 0.87	0.38
PC 16:0_20:3	− 5.72E−6	− 2.02E−5 to 6.64E−6	− 0.88	0.38
PC 18:0_18:1	− 1.65E−6	− 2.11E−5 to 1.57E−5	− 0.18	0.85
PC 18:0_20:3	− 1.83E−5	− 7.98E−5 to − 1.43E−5	− 1.95	0.05
SM d18:1/22:0	− 4.23E−5	− 7.98E−5 to − 1.43E−5	− 2.63	0.01
SM d18:1/22:1	− 2.78E−5	− 6.16E−5 to − 6.22E−6	− 1.97	0.05
SM d18:1/24:0	− 9.91E−6	− 3.79E−5 to 1.36E−5	− 0.79	0.43
TG 16:0_16:1_18:1	− 3.22E−6	− 1.57E−5 to 7.93E−6	− 0.57	0.57

**Table 4:** Coefficients for the logistic regression of diagnostic model “control group/RUF group”.

	β	CI β	Z stat	p
Free coefficient	− 1.24E1	− 2.33E1 to − 4.82E0		
PC 16:0_22:6	2.09E−6	4.36E−7 to 4.10E−6	2.28	0.02
PC 16:0_18:2	2.74E−6	1.08E−6 to 6.0E−6	2.32	0.02
PC 16:0_20:3	− 1.21E−6	− 9.00E−6 to 4.16E−6	− 0.39	0.70
PC 18:0_20:3	8.17E−6	− 4.36E−6 to 2.30E−5	1.21	0.23
SM d12:0/14:1	5.32E−6	2.17E−6 to 1.03E−5	2.64	0.01
SM d18:1/24:1	− 5.74E−6	− 1.75E−5 to 5.08E−6	− 1.02	0.31
SM d18:2/24:1	2.87E−5	2.14E−6 to 6.03E−5	1.99	0.05
PC 18:0_18:1	− 1.61E−5	− 3.07E−5 to − 3.74E−6	− 2.39	0.02

**Figure 4:**
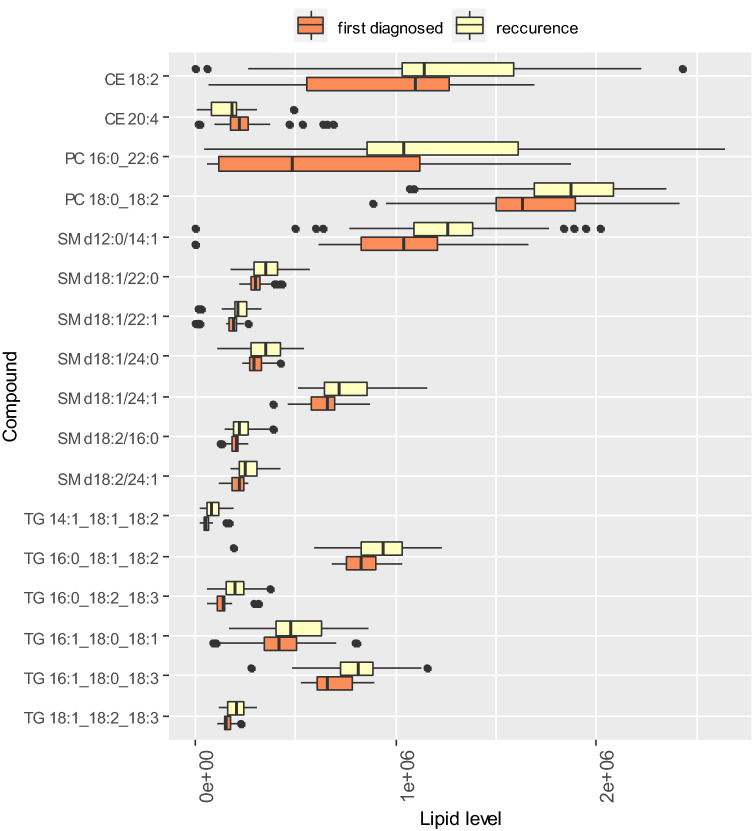
Relative intensity of marker plasma lipids in the OPLS-DA model classifying UF and RUF groups. Orange color corresponds to first-time diagnosed UF, and yellow color corresponds to the RUF. The diagram shows Q1 − 1.5 × IQR, Q1, Me, Q3, Q3 + 1.5 × IQR. Black dots correspond to outliers.

Lipid species identified as potentially significant in blood plasma for the differentiation between first-time UF and RUF include cholesterol esters, phosphotidylcholines, sphingomyelins and triglycerides (Table 2, Fig. 4).

High triglyceride levels may indirectly indicate the role of obesity in the development of UF and RUF. Obesity is a chronic disease of major public health concern. Earlier studies indicated several mechanisms that may promote the development of UFs in pre-menopausal women with excessive body fat. Firstly, high level of estrogens from body fat is partially responsible for UFs cells proliferation. Secondly, decrease in sex hormone binding globulin hepatic synthesis raises the level of active estrogens in blood. Finally, obesity is associated with significant systemic inflammation resulting in excessive reactive oxygen production^72,73^.

Several sphingomyelins (SM d12:0/14:1, SM d18:1/22:0, SM d18:1/22:1, SM d18:1/24:0, SM d18:1/24:1, SM d18:2/16:0, SM d18:2/24:1), were observed at higher concentrations in the plasma of RUF patients. Sphingomyelins are key components of the sphingomyelin cycle signal transduction pathway. Some metabolites of the sphingomyelin cycle, including ceramide and sphingosine, have been previously reported to induce apoptosis, while sphingosine-1-phosphate (S-1P) has been reported to promote cell survival in response to apoptotic stimuli^67^. Partial physiological consequences of this process could be the suppression of apoptosis in RUF patients on the cellular level (uterine fibroids), as well on systemic level (plasma)”.

The choice of treatment, including the degree of surgical treatment, depends upon the patient’s desire regarding the reproductive function. The revealed lipid panels may indicate a high risk of recurrence of the disease and, accordingly, the need for repeated surgical intervention. The patient would have to be informed about this, with additional reference to the anamnestic data. Moreover, the developed mathematical models make it possible to inform the patient whether or not the implementation of the reproductive function would be needed right after surgical treatment, without delay. Note that this is the first study to characterize the lipid profile of blood plasma in patients with UF and RUF. However, it is necessary to mention that the number of patients in this study was relatively small. Larger number of tissue samples should be analyzed to confirm the results obtained in this pilot study and to introduce the obtained panels into practical health care. The groups studied were not divided into genetic subtypes. The study of different genetic subtypes could be important to better understand the pathogenesis of UF, explaining the processes of development and recurrence of UF and tumor growth rates^53,74^. To identify a clinically meaningful biomarker of fibroid recurrence, this should be detected in fibroids removed from a patient that later on shows fibroid recurrence. Unfortunately, in this study we could not conduct such an experiment due to the lack of material from the same patients in our biobank. UF recurrence can occur within many years, which makes sample collection from RUF patients very difficult. Here, we conducted our prognostic study, which showed the presence of significant biomarkers of fibroid recurrence. The recruitment of patients for a more complete study is underway to conduct a complete study and compare the biomarkers with those discussed in this study.

## Conclusions

Mass spectrometry metabolic profiling of blood plasma and/or endometrium before surgical treatment will increase the efficiency of the operation, reduce the risk of recurrence and improve reproductive outcomes.

The results of our comparative study of lipid profiles in blood plasma, UF tissues and myometrium tissues suggest new potential molecular markers for the prediction of UF recurrence. The greatest changes in the lipid composition associated with the UF recurrence were observed in the UF tumor tissue. The level of 20 lipid species showed significant changes both in the myometrium and in the myomatous nodes during the UF recurrence. For 19 out of the 20 differential lipid species the increase in expression was found. In fibroids and myometrium samples, alterations in the level of lipids related to the metabolism of glycerophospholipids and sphingolipids were prominent. In fibroids tissues, linoleic acid metabolism was also notably altered. A number of phospholipids, sphingomyelins, cholesterol esters and triglycerides displayed significantly different levels in blood plasma of women with UF, RUF and the control group. Diagnostic models based on the selected lipids using logistic regression show sensitivity and specificity of 88% and 86% for the diagnosis of first-time UF and 95% and 79% for RUF.

These results indicate the potential of the lipid profiling of blood plasma for the low-invasive diagnosis of fibroids. Determination of significant molecular alterations in the tissues of fibroids will make it possible to give recommendations regarding further treatment, rehabilitation and reproductive function correction. Further study of molecular processes in the myometrium and in the fibroids as well as the determination of the ratio of proliferation and apoptosis processes will enhance our mechanistic understanding of UF and its recurrence.

## Supplementary Information

Supplementary Information.

## References

[1] Genazzani AD, Chierchia E, Despini G, Prati A (2016). Medical treatment of myomas. Front. Gynecol. Endocrinol..

[2] Al-Hendy A, Myers ER, Stewart E (2017). Uterine fibroids: Burden and unmet medical need. Semin. Reprod. Med..

[3] Donnez J, Donnez O, Dolmans MM (2014). With the advent of selective progesterone receptor modulators, what is the place of myoma surgery in current practice?. Fertil. Steril..

[4] American Association of Gynecologic Laparoscopists (AAGL) (2012). AAGL practice report: Practice guidelines for the diagnosis and management of submucous leiomyomas. J. Minim. Invasive Gynecol..

[5] Vilos GA (2015). The management of uterine leiomyomas. J. Obstet. Gynaecol. Can..

[6] Stewart EA, Cookson CL, Gandolfo RA, Schulze-Rath R (2017). Epidemiology of uterine fibroids: A systematic review. BJOG Int. J. Obstet. Gynaecol..

[7] Pérez-López FR (2014). EMAS position statement: Management of uterine fibroids. Maturitas.

[8] De La Cruz MS, Buchanan EM (2017). Uterine fibroids: Diagnosis and treatment. Am. Fam. Phys..

[9] Mas A (2017). Updated approaches for management of uterine fibroids. Int. J. Womens Health.

[10] Mäkinen N (2011). MED12, the mediator complex subunit 12 gene, is mutated at high frequency in uterine leiomyomas. Science.

[11] Markowski DN (2011). HMGA2 and p14Arf: Major roles in cellular senescence of fibroids and therapeutic implications. Anticancer Res..

[12] Tal R, Segars JH (2014). The role of angiogenic factors in fibroid pathogenesis: Potential implications for future therapy. Hum. Reprod. Update.

[13] Ren Y (2011). Different effects of epidermal growth factor on smooth muscle cells derived from human myometrium and from leiomyoma. Fertil. Steril..

[14] Torres-de la Roche LA (2017). Pathobiology of myomatosis uteri: The underlying knowledge to support our clinical practice. Arch. Gynecol. Obstet..

[15] Plewka D, Morek M, Bogunia E, Waloszek J, Plewka A (2016). Expression of VEGF isoforms and their receptors in uterine myomas. Ginekol. Pol..

[16] Baird DD, Dunson DB, Hill MC, Cousins D, Schectman JM (2003). High cumulative incidence of uterine leiomyoma in black and white women: Ultrasound evidence. Am. J. Obstet. Gynecol..

[17] Chiaffarino F (2017). Alcohol consumption and risk of uterine myoma: A systematic review and meta analysis. PLoS ONE.

[18] Rothmund R (2013). Clinical and pathological characteristics, pathological reevaluation and recurrence patterns of cellular leiomyomas: A retrospective study in 76 patients. Eur. J. Obstet. Gynecol. Reprod. Biol..

[19] Rafnar T (2018). Variants associating with uterine leiomyoma highlight genetic background shared by various cancers and hormone-related traits. Nat. Commun..

[20] Commandeur AE, Styer AK, Teixeira JM (2015). Epidemiological and genetic clues for molecular mechanisms involved in uterine leiomyoma development and growth. Hum. Reprod. Update.

[21] Nishiyama S (2006). High recurrence rate of uterine fibroids on transvaginal ultrasound after abdominal myomectomy in Japanese women. Gynecol. Obstet. Investig..

[22] Filho WMNE (2019). Evaluation of KI-67 expression in uterine leiomyoma and in healthy myometrium: A pilot study. Rev. Assoc. Med. Bras..

[23] Zhang D, Liu E (2018). Expression and clinical significance of VEGF, miR-18a and MCM7 in uterus myoma tissues. J. Hebei Med. Univ..

[24] Pascual Botia C, Camarasa SC, Raga Baixauli F, Sanchez AC (2017). Uterine fibroids review: Understanding their origins to better understand their future treatments. J. Tumor Res..

[25] Gracia M, Carmona F (2020). Uterine myomas: Clinical impact and pathophysiological bases. Eur. J. Obstet. Gynecol. Reprod. Biol..

[26] Rochat B (2016). From targeted quantification to untargeted metabolomics: Why LC-high-resolution-MS will become a key instrument in clinical labs. Trends Anal. Chem..

[27] Chagovets V (2018). A comparison of tissue spray and lipid extract direct injection electrospray ionization mass spectrometry for the differentiation of eutopic and ectopic endometrial tissues. J. Am. Soc. Mass Spectrom..

[28] Chagovets VV (2017). Endometriosis foci differentiation by rapid lipid profiling using tissue spray ionization and high resolution mass spectrometry. Sci. Rep..

[29] Cífková E (2015). Determination of lipidomic differences between human breast cancer and surrounding normal tissues using HILIC-HPLC/ESI-MS and multivariate data analysis. Anal. Bioanal. Chem..

[30] Jarmusch AK (2016). Differential Lipid profiles of normal human brain matter and gliomas by positive and negative mode desorption electrospray ionization—Mass spectrometry imaging. PLoS ONE.

[31] Jiang Y (2013). Altered sphingolipid metabolism in patients with metastatic pancreatic cancer. Biomolecules.

[32] Kang S (2011). Alteration in lipid and protein profiles of ovarian cancer similarity to breast cancer. Int. J. Gynecol. Cancer.

[33] Ishikawa S (2012). Increased expression of phosphatidylcholine (16:0/18:1) and (16:0/18:2) in thyroid papillary cancer. PLoS ONE.

[34] Tokareva AO (2020). Feature selection for OPLS discriminant analysis of cancer tissue lipidomics data. J. Mass Spectrom..

[35] Sans M (2017). Metabolic markers and statistical prediction of serous ovarian cancer aggressiveness by ambient ionization mass spectrometry imaging. Cancer Res..

[36] Kim IC (2013). Lipid profiles for HER2-positive breast cancer. Anticancer Res..

[37] Chagovets V (2019). Relative quantitation of phosphatidylcholines with interfered masses of protonated and sodiated molecules by tandem and Fourier-transform ion cyclotron resonance mass spectrometry. Eur. J. Mass Spectrom..

[38] Kwon SY (2014). Lipid MALDI MS profiles of gastric cancer. Open Proteomics J..

[39] Zhao X (2018). Lipidomic profiling links the fanconi anemia pathway to glycosphingolipid metabolism in head and neck cancer cells. Clin. Cancer Res..

[40] Chagovets V (2016). Peculiarities of data interpretation upon direct tissue analysis by Fourier transform ion cyclotron resonance mass spectrometry. Eur. J. Mass Spectrom..

[41] Kononikhin A (2015). A novel direct spray-from-tissue ionization method for mass spectrometric analysis of human brain tumors. Anal. Bioanal. Chem..

[42] Kim IC (2016). Erratum: Low C24-OH and C22-OH sulfatides in human renal cell carcinoma (Journal of Mass Spectrometry (2014) 49 (409–416)). J. Mass Spectrom..

[43] Morita Y (2013). Lysophosphatidylcholine acyltransferase 1 altered phospholipid composition and regulated hepatoma progression. J. Hepatol..

[44] Chagovets VV (2020). Validation of breast cancer margins by tissue spray mass spectrometry. Int. J. Mol. Sci..

[45] Altadill T (2017). Metabolomic and lipidomic profiling identifies the role of the RNA editing pathway in endometrial carcinogenesis. Sci. Rep..

[46] Wei Y (2015). Tissue spray ionization mass spectrometry for rapid recognition of human lung squamous cell carcinoma. Sci. Rep..

[47] Starodubtseva N (2019). Identification of potential endometriosis biomarkers in peritoneal fluid and blood plasma via shotgun lipidomics. Clin. Mass Spectrom..

[48] Li J (2018). Discovery of phosphatidic acid, phosphatidylcholine, and phosphatidylserine as biomarkers for early diagnosis of endometriosis. Front. Physiol..

[49] Lee GK (2012). Lipid MALDI profile classifies non-small cell lung cancers according to the histologic type. Lung Cancer.

[50] Sukhikh G (2019). Combination of low-temperature electrosurgical unit and extractive electrospray ionization mass spectrometry for molecular profiling and classification of tissues. Molecules.

[51] Porcari AM (2018). Molecular signatures of high-grade cervical lesions. Front. Oncol..

[52] Adamyan LV (2018). Direct mass spectrometry differentiation of ectopic and eutopic endometrium in patients with endometriosis. J. Minim. Invasive Gynecol..

[53] Heinonen HR (2017). Global metabolomic profiling of uterine leiomyomas. Br. J. Cancer.

[54] Heinonen HR (2017). Multiple clinical characteristics separate MED12-mutation-positive and -negative uterine leiomyomas. Sci. Rep..

[55] Folch J, Lees M, Sloane Stanley GH (1957). A simple method for the isolation and purification of total lipides from animal tissues. J. Biol. Chem..

[56] Koelmel JP (2017). LipidMatch: An automated workflow for rule-based lipid identification using untargeted high-resolution tandem mass spectrometry data. BMC Bioinform..

[57] Sud M (2007). LMSD: LIPID MAPS structure database. Nucleic Acids Res..

[58] Vabalas A, Gowen E, Poliakoff E, Casson AJ (2019). Machine learning algorithm validation with a limited sample size. PLoS ONE.

[59] Li J (2017). Distinct plasma lipids profiles of recurrent ovarian cancer by liquid chromatography-mass spectrometry. Oncotarget.

[60] Gorden DL (2015). Biomarkers of NAFLD progression: A lipidomics approach to an epidemic. J. Lipid Res..

[61] Islam MS (2018). Omega-3 fatty acids modulate the lipid profile, membrane architecture, and gene expression of leiomyoma cells. J. Cell. Physiol..

[62] Nagan N, Zoeller RA (2001). Plasmalogens: Biosynthesis and functions. Prog. Lipid Res..

[63] Wang X, Devaiah SP, Zhang W, Welti R (2006). Signaling functions of phosphatidic acid. Prog. Lipid Res..

[64] Chen J, Ahmed R (2004). Novel regulatory mechanisms of mTOR signaling. Current Topics in Microbiology and Immunology.

[65] O’Luanaigh N (2002). Continual production of phosphatidic acid by phospholipase D is essential for antigen-stimulated membrane ruffling in cultured. Mol. Biol. Cell.

[66] Su W, Chardin P, Yamazaki M, Kanaho Y, Du G (2006). RhoA-mediated phospholipase D1 signaling is not required for the formation of stress fibers and focal adhesions. Cell. Signal..

[67] Cuvillier O (2002). Sphingosine in apoptosis signaling. Biochim. Biophys. Acta Mol. Cell Biol. Lipids.

[68] Arana L, Gangoiti P, Ouro A, Trueba M, Gómez-Muñoz A (2010). Ceramide and ceramide 1-phosphate in health and disease. Lipids Health Dis..

[69] van der Veen JN (2017). The critical role of phosphatidylcholine and phosphatidylethanolamine metabolism in health and disease. Biochim. Biophys. Acta Biomembr..

[70] Ridgway ND (2013). The role of phosphatidylcholine and choline metabolites to cell proliferation and survival. Crit. Rev. Biochem. Mol. Biol..

[71] Segawa K, Nagata S (2015). An apoptotic ‘Eat Me’ signal: Phosphatidylserine exposure. Trends Cell Biol..

[72] Maggio M (2008). Sex hormones binding globulin levels across the adult lifespan in women—The role of body mass index and fasting insulin. J. Endocrinol. Investig..

[73] Soave I, Marci R (2018). From obesity to uterine fibroids: An intricate network. Curr. Med. Res. Opin..

[74] Mäkinen N (2013). MED12 exon 2 mutations in histopathological uterine leiomyoma variants. Eur. J. Hum. Genet..

