# Supplementary material for: Alterations in lipid profile upon uterine fibroids and its recurrence
Source: Sci Rep. 2021 Jun 1;11:11447. doi: 10.1038/s41598-021-89859-0 (PMC8169782; doi:10.1038/s41598-021-89859-0)
Supplement: Supplementary file 1 — Supplementary Information. [file 41598_2021_89859_MOESM1_ESM.docx]

**Supplementary**

**Alterations in lipid profile upon uterine fibroids and its recurrence**

Narine M. Tonoyan^1^, Vitaliy V. Chagovets^1^, Natalia L. Starodubtseva*^1,3^*, Alisa O. Tokareva^1,2^, Konstantin Chingin^4^, Irena F. Kozachenko^1^, Leyla V. Adamyan^1^, Vladimir E. Frankevich^1^*^*^*

^1^ National Medical Research Center for Obstetrics, Gynecology and Perinatology named after Academician V.I. Kulakov of the Ministry of Healthcare of Russian Federation, Moscow 117997, Russian Federation;

^2^ V.L. Talrose Institute for Energy Problems of Chemical Physics, Russia Academy of Sciences, Moscow 119991, Russian Federation;

^3^ Moscow Institute of Physics and Technology, Moscow region, 141700, Russian Federation

^4^Jiangxi Key Laboratory for Mass Spectrometry and Instrumentation, East China University of Technology, Nanchang 330013, China

*Correspondence: vfrankevich@gmail.com

**
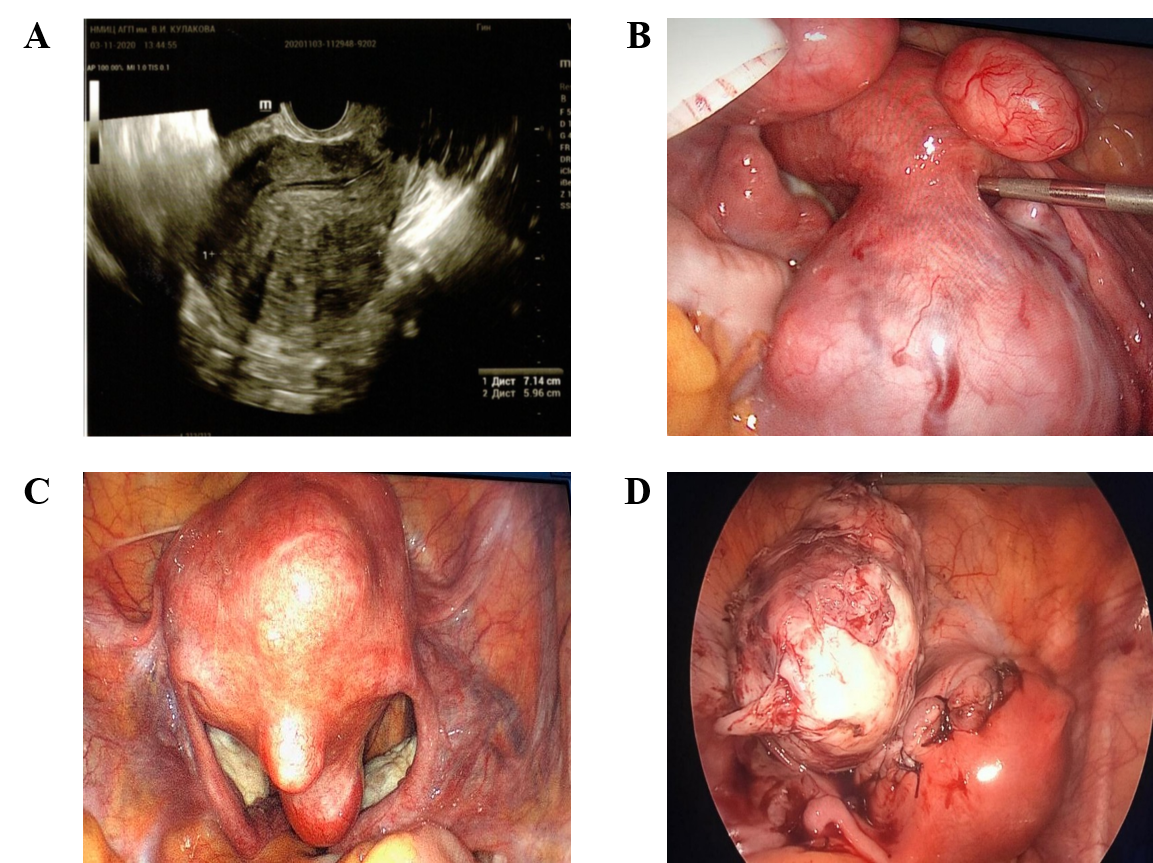
**Figure 1S. The example of ultrasound examination (A) and laparoscopy procedure (C-D) in UF case.


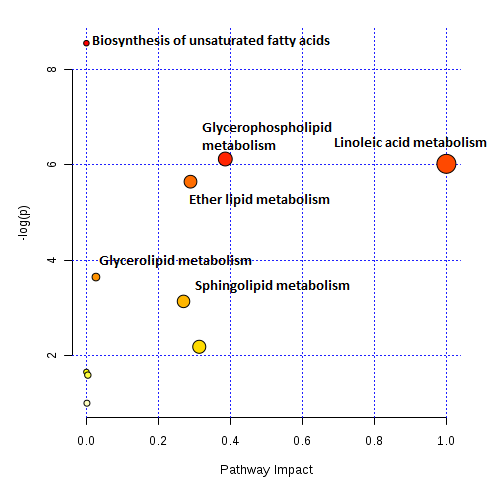


Figure 2S. Diagram of metabolic pathways for uterine fibroids lipids significantly changed in UF group compared to RUF.

Figure 3S. Relative intensity of marker plasma lipids in OPLS-DA model classifying UF and control groups. Yellow color corresponds to first-time diagnosed fibroids (UF). Orange color corresponds to control group. The diagram shows Q1–1.5*IQR, Q1, Me, Q3, Q3+1.5*IQR. Black dots correspond to outliers.

Figure 4S. Relative intensity of marker plasma lipids in OPLS-DA model classifying RUF and control groups. Yellow color corresponds to recurrence of uterine fibroids (RUF). Orange color corresponds to control group. The diagram shows Q1–1.5*IQR, Q1, Me, Q3, Q3+1.5*IQR. Black dots correspond to outliers.

Table 1S. Myometrium lipid species with statistically significant differences (p<0.05) between UF and RUF clinical groups. Relative levels are presented in Me (Q1, Q3).

| Lipid species | UF relative level | RUF relative level | Fold change, % | p-value |
| --- | --- | --- | --- | --- |
| Cer 34:2 | 3.5e+03(1.3e+03;7.3e+03) | 1.1e+04(2.9e+03;1.7e+04) | 314.29 | 0.004 |
| Cer 40:3 | 1.8e+02(1.1e+02;2.9e+02) | 2.8e+02(1.7e+02;5.1e+02) | 155.56 | 0.03 |
| FA 16:0 | 9.6e+04(7.5e+04;1.5e+05) | 1.4e+05(1.2e+05;1.7e+05) | 145.83 | 0.008 |
| FA 20:1 | 2.2e+03(1.2e+03;4.2e+03) | 4.8e+03(3.1e+03;6.6e+03) | 218.18 | 0.01 |
| FA 20:2 | 5.5e+03(2.0e+03;1.1e+04) | 1.2e+04(7.3e+03;1.7e+04) | 218.18 | 0.004 |
| FA 20:3 | 7.9e+04(3.8e+04;1.0e+05) | 1.2e+05(6.9e+04;1.6e+05) | 151.90 | 0.03 |
| FA 20:4 | 4.2e+05(2.0e+05;8.9e+05) | 8.4e+05(5.8e+05;1.1e+06) | 200.00 | 0.02 |
| FA 20:5 | 6.8e+03(2.5e+03;8.5e+03) | 1.0e+04(4.0e+03;1.8e+04) | 147.06 | 0.04 |
| FA 22:1 | 4.1e+02(2.6e+02;6.0e+02) | 8.0e+02(4.2e+02;1.5e+03) | 195.12 | 0.01 |
| FA 22:3 | 6.9e+02(2.4e+02;1.2e+03) | 1.5e+03(7.7e+02;2.3e+03) | 217.39 | 0.009 |
| FA 22:4 | 2.5e+04(1.4e+04;4.9e+04) | 5.0e+04(3.8e+04;6.4e+04) | 200.00 | 0.005 |
| FA 22:5 | 1.7e+04(9.2e+03;3.0e+04) | 3.6e+04(1.9e+04;4.7e+04) | 211.76 | 0.003 |
| LPC 16:0 | 8.9e+04(6.8e+04;1.3e+05) | 4.8e+04(3.2e+04;1.0e+05) | 53.93 | 0.04 |
| LPC 16:1 | 6.5e+02(4.7e+02;9.0e+02) | 2.7e+02(1.5e+02;4.8e+02) | 41.54 | 0.01 |
| LPC 18:2 | 1.9e+05(1.5e+05;2.3e+05) | 1.3e+05(6.2e+04;2.1e+05) | 68.42 | 0.04 |
| LPC O-16:0 | 1.8e+04(9.6e+03;2.8e+04) | 8.9e+03(4.2e+03;1.9e+04) | 49.44 | 0.04 |
| PA 44:5 | 1.3e+06(7.2e+05;1.8e+06) | 1.7e+06(1.3e+06;2.1e+06) | 130.77 | 0.04 |
| PA 48:0 | 5.8e+01(4.7e+01;1.0e+02) | 1.3e+02(5.9e+01;3.3e+02) | 224.14 | 0.004 |
| PC 40:0 | 3.5e+02(2.2e+02;5.5e+02) | 7.1e+02(3.7e+02;3.8e+03) | 202.86 | 0.009 |
| PC 40:5 | 8.7e+05(7.7e+05;1.7e+06) | 1.6e+06(1.3e+06;2.2e+06) | 183.91 | 0.009 |
| PC O-32:0 | 6.7e+02(3.1e+02;9.9e+02) | 1.7e+03(8.6e+02;2.6e+03) | 253.73 | 0.004 |
| PC O-38:0 | 1.7e+02(1.1e+02;2.2e+02) | 3.7e+02(2.2e+02;1.3e+03) | 217.65 | 0.002 |
| PE 38:3 | 1.1e+03(0.0e+00;2.0e+03) | 2.2e+03(4.3e+02;9.0e+04) | 200.00 | 0.03 |
| PE 38:4 | 1.5e+06(6.4e+05;1.6e+06) | 9.4e+05(3.0e+05;1.3e+06) | 62.67 | 0.04 |
| PE 40:4 | 1.2e+04(3.4e+03;1.7e+04) | 3.0e+04(9.3e+03;6.2e+04) | 250.00 | 0.001 |
| PE 40:5 | 1.6e+04(3.9e+03;2.9e+04) | 4.0e+04(8.6e+03;7.6e+04) | 250.00 | 0.01 |
| PE 42:4 | 1.7e+02(1.1e+02;2.3e+02) | 2.3e+02(1.6e+02;3.5e+02) | 135.29 | 0.04 |
| PE O-38:3 | 1.3e+03(6.3e+02;2.4e+03) | 3.3e+03(9.9e+02;8.9e+03) | 253.85 | 0.04 |
| PE O-38:4 | 7.4e+02(0.0e+00;1.3e+03) | 4.3e+03(2.0e+02;8.4e+04) | 581.08 | 0.03 |
| PS 38:1 | 8.3e+02(4.4e+02;1.1e+03) | 2.0e+03(6.0e+02;4.2e+03) | 240.96 | 0.02 |
| PS 40:3 | 1.2e+03(9.9e+02;1.5e+03) | 6.8e+02(3.7e+02;1.4e+03) | 56.67 | 0.03 |
| PS 42:6 | 6.0e+02(4.2e+02;8.4e+02) | 1.5e+03(6.2e+02;2.6e+03) | 250.00 | 0.01 |
| PS 46:1 | 2.1e+02(1.1e+02;3.1e+02) | 3.8e+02(1.4e+02;1.4e+03) | 180.95 | 0.04 |
| SM 32:1 | 1.6e+05(8.9e+04;2.1e+05) | 9.0e+04(5.6e+04;1.5e+05) | 56.25 | 0.04 |
| TG 30:0 | 6.9e+03(7.4e+02;2.0e+04) | 9.8e+02(4.6e+02;4.2e+03) | 14.20 | 0.04 |
| TG 42:1 | 6.3e+02(5.1e+02;1.3e+03) | 1.1e+03(8.5e+02;1.9e+03) | 174.60 | 0.03 |
| TG 56:8 | 9.6e+04(7.5e+04;1.4e+05) | 1.3e+05(9.1e+04;2.3e+05) | 135.42 | 0.03 |

Table 2S. Uterine fibroids lipid species with statistically significant differences (p<0.05) between UF and RUF clinical groups. Relative levels are presented in Me (Q1, Q3).

| Lipid species | UF relative level | RUF relative level | Fold change, % | p-value |
| --- | --- | --- | --- | --- |
| Cer 34:2 | 2.4e+03(1.4e+03;5.4e+03) | 9.4e+03(4.6e+03;2.1e+04) | 391.67 | 0.002 |
| Cer 36:2 | 7.4e+02(2.6e+02;1.9e+03) | 2.4e+03(1.3e+03;6.5e+03) | 324.32 | 0.009 |
| Cer 36:3 | 5.1e+01(4.5e+01;6.0e+01) | 7.3e+01(5.1e+01;9.2e+01) | 143.14 | 0.04 |
| Cer 38:2 | 5.7e+02(3.1e+02;9.3e+02) | 9.1e+02(6.2e+02;4.7e+03) | 159.65 | 0.01 |
| Cer 38:3 | 6.2e+01(5.7e+01;8.3e+01) | 1.2e+02(6.5e+01;1.5e+02) | 193.55 | 0.02 |
| Cer 40:2 | 6.1e+03(2.5e+03;7.6e+03) | 1.1e+04(5.1e+03;2.7e+04) | 180.33 | 0.04 |
| FA 18:1 | 1.9e+05(1.2e+05;2.1e+05) | 2.3e+05(1.6e+05;3.3e+05) | 121.05 | 0.04 |
| FA 18:2 | 8.6e+04(6.4e+04;1.3e+05) | 1.4e+05(9.3e+04;1.9e+05) | 162.79 | 0.01 |
| FA 20:1 | 2.9e+03(1.5e+03;4.9e+03) | 6.7e+03(3.0e+03;1.4e+04) | 231.03 | 0.01 |
| FA 20:2 | 8.9e+03(6.4e+03;1.4e+04) | 2.2e+04(1.1e+04;4.0e+04) | 247.19 | 0.004 |
| FA 20:3 | 8.0e+04(4.4e+04;9.0e+04) | 1.2e+05(8.8e+04;1.8e+05) | 150.00 | 0.002 |
| FA 20:4 | 3.9e+05(2.9e+05;6.3e+05) | 7.3e+05(5.3e+05;8.7e+05) | 187.18 | 0.003 |
| FA 20:5 | 5.4e+03(1.8e+03;7.0e+03) | 1.0e+04(5.2e+03;1.4e+04) | 185.19 | 0.02 |
| FA 22:1 | 5.5e+02(2.8e+02;1.1e+03) | 1.5e+03(5.2e+02;3.1e+03) | 272.73 | 0.04 |
| FA 22:2 | 2.7e+02(1.5e+02;7.8e+02) | 1.2e+03(4.7e+02;3.4e+03) | 444.44 | 0.004 |
| FA 22:3 | 1.0e+03(8.0e+02;3.5e+03) | 4.7e+03(2.2e+03;1.1e+04) | 470.00 | 0.006 |
| FA 22:4 | 4.5e+04(2.3e+04;6.1e+04) | 8.7e+04(5.5e+04;1.2e+05) | 193.33 | 0.004 |
| FA 22:5 | 2.1e+04(9.8e+03;3.2e+04) | 4.0e+04(2.7e+04;7.0e+04) | 190.48 | 0.003 |
| FA 22:6 | 2.2e+04(1.4e+04;2.8e+04) | 5.6e+04(4.0e+04;8.2e+04) | 254.55 | <0.001 |
| FA 24:1 | 1.2e+03(6.7e+02;1.6e+03) | 4.3e+03(1.9e+03;8.4e+03) | 358.33 | 0.003 |
| LPE O-18:1 | 7.9e+02(4.5e+02;1.7e+03) | 3.0e+03(1.4e+03;8.1e+03) | 379.75 | 0.02 |
| PA 44:5 | 1.5e+06(6.1e+05;2.1e+06) | 2.3e+06(1.7e+06;3.1e+06) | 153.33 | 0.02 |
| PA 44:6 | 5.3e+05(1.7e+05;8.7e+05) | 8.5e+05(7.0e+05;1.3e+06) | 160.38 | 0.03 |
| PC 32:3 | 7.6e+04(5.9e+04;1.3e+05) | 5.0e+04(3.6e+04;8.7e+04) | 65.79 | 0.02 |
| PC 34:0 | 1.3e+07(9.8e+06;1.7e+07) | 1.8e+07(1.4e+07;2.0e+07) | 138.46 | 0.02 |
| PC 34:1 | 8.5e+07(7.0e+07;1.2e+08) | 1.4e+08(1.1e+08;1.6e+08) | 164.71 | 0.004 |
| PC 36:1 | 3.0e+07(2.0e+07;3.6e+07) | 3.7e+07(2.7e+07;4.8e+07) | 123.33 | 0.04 |
| PC 36:2 | 4.3e+07(3.4e+07;5.4e+07) | 6.3e+07(4.9e+07;7.9e+07) | 146.51 | 0.003 |
| PC 36:4 | 3.6e+07(3.2e+07;5.6e+07) | 5.7e+07(4.2e+07;6.5e+07) | 158.33 | 0.04 |
| PC 38:2 | 1.4e+06(6.1e+05;2.1e+06) | 2.4e+06(1.2e+06;3.0e+06) | 171.43 | 0.04 |
| PC 46:0 | 2.3e+04(1.9e+03;4.4e+04) | 1.1e+03(7.4e+02;1.5e+03) | 4.78 | 0.002 |
| PC 48:5 | 2.8e+03(8.4e+02;6.9e+03) | 7.1e+02(4.8e+02;3.1e+03) | 25.36 | 0.04 |
| PC O-32:0 | 4.1e+05(1.8e+04;6.8e+05) | 7.3e+05(3.7e+05;9.1e+05) | 178.05 | 0.04 |
| PC O-34:3 | 9.7e+04(6.3e+04;1.9e+05) | 2.3e+05(1.4e+05;5.4e+05) | 237.11 | 0.005 |
| PC O-38:4 | 6.4e+05(2.7e+05;1.0e+06) | 1.2e+06(7.2e+05;1.9e+06) | 187.50 | 0.006 |
| PC O-40:4 | 4.7e+03(2.5e+03;1.6e+04) | 2.0e+04(6.3e+03;4.0e+04) | 425.53 | 0.04 |
| PC O-40:6 | 1.1e+03(5.5e+02;2.4e+03) | 2.3e+03(1.2e+03;5.7e+03) | 209.09 | 0.04 |
| PE 38:2 | 8.7e+03(1.0e+03;1.6e+04) | 1.5e+04(5.9e+03;2.2e+04) | 172.41 | 0.04 |
| PE 40:4 | 1.2e+04(2.7e+03;2.7e+04) | 3.3e+04(1.5e+04;6.5e+04) | 275.00 | 0.02 |
| PE 40:5 | 2.5e+04(8.5e+03;3.5e+04) | 4.4e+04(2.8e+04;8.5e+04) | 176.00 | 0.01 |
| PE 42:4 | 1.6e+02(8.4e+01;2.2e+02) | 2.3e+02(1.5e+02;3.8e+02) | 143.75 | 0.02 |
| PE 42:5 | 3.0e+02(2.0e+02;5.1e+02) | 4.6e+02(3.4e+02;7.7e+02) | 153.33 | 0.04 |
| PE O-36:5 | 2.5e+06(2.3e+06;3.7e+06) | 4.0e+06(3.0e+06;4.9e+06) | 160.00 | 0.02 |
| PE O-38:5 | 4.8e+06(3.0e+06;5.9e+06) | 6.5e+06(3.5e+06;8.9e+06) | 135.42 | 0.04 |
| PE O-38:6 | 1.4e+06(1.2e+06;3.1e+06) | 2.3e+06(2.0e+06;3.4e+06) | 164.29 | 0.04 |
| PS 38:3 | 2.4e+04(1.5e+04;4.4e+04) | 7.7e+03(1.8e+03;1.9e+04) | 32.08 | 0.03 |
| PS 40:3 | 1.3e+03(5.9e+02;1.9e+03) | 5.4e+02(1.9e+02;8.4e+02) | 41.54 | 0.008 |
| SM 34:1 | 2.6e+07(2.2e+07;3.1e+07) | 3.3e+07(2.8e+07;4.1e+07) | 126.92 | 0.003 |
| SM 36:1 | 2.4e+06(1.7e+06;3.0e+06) | 3.5e+06(2.7e+06;4.6e+06) | 145.83 | 0.005 |
| SM 38:1 | 5.2e+05(2.6e+05;7.0e+05) | 7.7e+05(5.1e+05;9.8e+05) | 148.08 | 0.03 |
| SM 42:1 | 3.0e+06(2.5e+06;3.3e+06) | 3.8e+06(3.0e+06;6.4e+06) | 126.67 | 0.004 |
| SM 42:2 | 9.8e+06(8.9e+06;1.4e+07) | 1.5e+07(1.2e+07;2.0e+07) | 153.06 | 0.005 |
| TG 30:0 | 1.1e+03(6.6e+02;2.1e+03) | 3.9e+02(3.4e+02;5.8e+02) | 35.45 | <0.001 |
| TG 36:0 | 2.4e+03(8.0e+02;5.4e+03) | 4.4e+02(3.5e+02;1.3e+03) | 18.33 | 0.002 |
| TG 38:0 | 9.7e+02(5.2e+02;5.0e+03) | 3.7e+02(2.4e+02;6.9e+02) | 38.14 | 0.003 |
| TG 40:0 | 9.8e+02(5.2e+02;2.3e+03) | 4.8e+02(2.5e+02;6.5e+02) | 48.98 | 0.002 |
| TG 42:0 | 5.0e+03(1.8e+03;9.5e+03) | 1.3e+03(8.1e+02;2.3e+03) | 26.00 | <0.001 |
| TG 42:1 | 2.3e+03(1.5e+03;4.7e+03) | 8.7e+02(6.5e+02;1.2e+03) | 37.83 | <0.001 |
| TG 44:0 | 5.2e+03(1.1e+03;1.3e+04) | 1.0e+03(7.1e+02;2.3e+03) | 19.23 | 0.02 |
| TG 44:1 | 3.8e+03(1.8e+03;1.3e+04) | 1.6e+03(1.3e+03;2.3e+03) | 42.11 | 0.004 |
| TG 44:2 | 8.7e+02(6.5e+02;2.2e+03) | 5.6e+02(4.7e+02;7.9e+02) | 64.37 | 0.008 |
| TG 46:0 | 3.6e+04(1.3e+04;1.4e+05) | 1.4e+04(5.1e+03;4.0e+04) | 38.89 | 0.04 |
| TG 46:1 | 2.6e+04(1.5e+04;8.3e+04) | 4.8e+03(2.4e+03;2.3e+04) | 18.46 | 0.02 |
| TG 46:2 | 1.0e+04(4.7e+03;3.0e+04) | 3.5e+03(1.5e+03;1.3e+04) | 35.00 | 0.02 |
| TG 48:4 | 1.3e+06(1.1e+06;2.0e+06) | 2.0e+06(1.3e+06;3.3e+06) | 153.85 | 0.02 |
| TG 58:10 | 2.9e+04(1.0e+04;1.3e+05) | 1.5e+04(3.9e+03;3.0e+04) | 51.72 | 0.04 |
